# Some Uses for “Dentalone.”

**Published:** 1910-12-15

**Authors:** C. S. Service

**Affiliations:** Philadelphia


### SOME USES FOR "DENTALONE."

BY C. S. SERVICE, D.D.S., PHILADELPHIA.

Dentalone is a combination of Chloretone and the essential oils of gaultheria cloves and cassia. It is anesthetic, disinfectant and antiseptic in action. As a local application in odontalgia, from caries or exposed pulps, and as a disinfectant and antiseptic dressing in root canals, and in combination with phosphoric acid in oxyphosphate fillings, to counteract the irritating effects of the acid on sensitive dentine, it is highly efficient. It is palliative and curative in its effects and is pleasant for the patient, having decided advantages over the unpleasant creosote, carbolic acid, iodoform, etc.

My methods of use are as follows; for odontalgia: after removing all debris and decayed tooth substance to the sensitive dentine, dry the cavity thoroughly and apply a small pledget of cotton saturated with Dentalone; next place a larger pledget of cotton over this to about half fill the cavity and seal with chlora-percha on cotton or a temporary stopping for three or four days, or longer, to a week. Then apply rubber dam and proceed with such treatment as may be indicated, frequently using a warm application of Dentalone to desensitize until the cavity is ready for filling.

If the cavity is deep and encroaches on the pulp, do not remove all the softened dentine over the pulp area, but saturate the cavity with Dentalone and make a capping of oxyphosphate filling. For this purpose take equal parts of Dentalone and phosphoric acid to make a creamy paste with the powder of such consistence that it will drop off the end of an instrument. Flow this mixture, which can be easily spread over the bottom of the cavity by means of a pledget of cotton dipped in the dry powder, and press it lightly over the floor of the cavity. Within two or three minutes it will be sufficiently hard to permit the addition of a thicker mixture of the same composition to half fill the cavity. Then use temporary stopping until the next sitting, when the filling can be completed with either a plastic or metal filling as the case indicates.

For pulpitis: before devitalizing the pulp apply Dentalone. Then dip the arsenical wafer or fibre in Dentalone before applying to the pulp. The anesthetic effect of the Chloretone is most gratifying, as the patient is unaware that anything unusual has taken place. Proceed in the usual manner of removing the pulp, using Dentalone on cotton strands for root canal treatments, and also use it in the same manner for disinfecting the root canals when the pulp has died and putrefactive gases have formed and decomposition of the pulp tissue has ensued.

For cavities located at the gingival margins or beneath the gum, where we find hypersensitive dentine, apply Dentalone as in odontalgia. Then make a mixture of equal parts of Dentalone and phosphoric acid with the oxyphosphate powder and make a temporary filling which I permit to remain six months or longer, when the sensitiveness is entirely overcome, and the cavity can be thoroughly prepared for a permanent filling without that excruciating pain so evident in that class of cavities. In all large cavities use the medicated cement as a foundation or lining to obviate the effects of thermal changes, so annoying in sensitive teeth. One other very good feature of Dentalone is that no one has ever complained of the taste, odor or effects of its use in the mouth, while with creosote and carbolic acid the taste and odor have been nauseating to many in the mouth, and in the atmosphere of the office.

